# Long-term ambient air pollution exposure and cardio-respiratory disease in China: findings from a prospective cohort study

**DOI:** 10.1186/s12940-023-00978-9

**Published:** 2023-03-27

**Authors:** Neil Wright, Katherine Newell, Ka Hung Chan, Simon Gilbert, Alex Hacker, Yan Lu, Yu Guo, Pei Pei, Canqing Yu, Jun Lv, Junshi Chen, Liming Li, Om Kurmi, Zhengming Chen, Kin Bong Hubert Lam, Christiana Kartsonaki

**Affiliations:** 1 Clinical Trial Service Unit and Epidemiological Studies Unit, Nuffield Department of Population Health, University of Oxford, Big Data Institute Building, Old Road Campus, OX3 7LF Oxford, UK; 2 Oxford British Heart Foundation Centre of Research Excellence, University of Oxford, Oxford, UK; 3 NCDs Prevention and Control Department, Suzhou CDC, Jiangsu, China; 4 Chinese Academy of Medical Sciences, Beijing, China; 5 Peking University Center for Public Health and Epidemic Preparedness and Response, Peking University, Beijing, China; 6 Department of Epidemiology and Biostatistics, School of Public Health, Peking University, Beijing, China; 7 National Center for Food Safety Risk Assessment, Beijing, China; 8 Research Centre for Intelligent Healthcare, Coventry University, Coventry, UK; 9 MRC Population Health Research Unit, University of Oxford, Oxford, UK

**Keywords:** Air pollution, Cardiovascular disease, Respiratory disease

## Abstract

**Background:**

Existing evidence on long-term ambient air pollution (AAP) exposure and risk of cardio-respiratory diseases in China is mainly on mortality, and based on area average concentrations from fixed-site monitors for individual exposures. Substantial uncertainty persists, therefore, about the shape and strength of the relationship when assessed using more personalised individual exposure data. We aimed to examine the relationships between AAP exposure and risk of cardio-respiratory diseases using predicted local levels of AAP.

**Methods:**

A prospective study included 50,407 participants aged 30–79 years from Suzhou, China, with concentrations of nitrogen dioxide (NO_2_), sulphur dioxide (SO_2_), fine (PM_2.5_), and inhalable (PM_10_) particulate matter, ozone (O_3_) and carbon monoxide (CO) and incident cases of cardiovascular disease (CVD) (*n* = 2,563) and respiratory disease (*n* = 1,764) recorded during 2013–2015. Cox regression models with time-dependent covariates were used to estimate adjusted hazard ratios (HRs) for diseases associated with local-level concentrations of AAP exposure, estimated using Bayesian spatio–temporal modelling.

**Results:**

The study period of 2013–2015 included a total of 135,199 person-years of follow-up for CVD. There was a positive association of AAP, particularly SO_2_ and O_3_, with risk of major cardiovascular and respiratory diseases. Each 10 µg/m^3^ increase in SO_2_ was associated with adjusted hazard ratios (HRs) of 1.07 (95% CI: 1.02, 1.12) for CVD, 1.25 (1.08, 1.44) for COPD and 1.12 (1.02, 1.23) for pneumonia. Similarly, each 10 µg/m^3^ increase in O_3_ was associated with adjusted HR of 1.02 (1.01, 1.03) for CVD, 1.03 (1.02, 1.05) for all stroke, and 1.04 (1.02, 1.06) for pneumonia.

**Conclusions:**

Among adults in urban China, long-term exposure to ambient air pollution is associated with a higher risk of cardio-respiratory disease.

**Supplementary Information:**

The online version contains supplementary material available at 10.1186/s12940-023-00978-9.

## Background

Ambient air pollution (AAP) is a major risk factor for many diseases, with ambient fine particulate matter (PM_2.5_) and ozone (O_3_) estimated to account for 4.5 million deaths worldwide in 2019, including 1.5 million in China [1]. These estimates were largely based on the dose–response relationships of ambient PM_2.5_ with cardio-respiratory diseases and O_3_ with chronic obstructive pulmonary disease (but not other conditions), derived from prospective cohort studies mostly conducted in western high-income populations. Meanwhile, the associations of other key air pollutants, such as sulphur dioxide (SO_2_), nitrogen dioxide (NO_2_), and carbon monoxide (CO) with health outcomes are less well-understood due to the lack of reliable prospective evidence [2].

In recent decades, China has undergone rapid economic growth, urbanisation, and industrialisation, with pollution levels far exceeding both international [3] and national guidelines [4]. While the short-term health effects of AAP exposure have been well-documented across China, especially for cardio-respiratory morbidity [5, 6] and mortality [7, 8], evidence is more limited on associations of AAP exposure with longer term health outcomes in China. For a few such studies in China, they mainly relied on spatially and temporally averaged concentrations from fixed-site monitors as proxies for personal exposure [9–14]. Recent studies have modelled exposure based on satellite remote sensing data, but at a relatively crude spatial and temporal resolution and/or for a limited number of pollutants [15–17]. We therefore aimed to examine the relationship between AAP exposure and cardio-respiratory disease incidence using exposure estimates from spatio–temporal modelling applied to individuals in a prospective Chinese cohort for all six criteria air pollutants.

## Methods

### Study design

We used data from the prospective China Kadoorie Biobank (CKB) study. Details of the study design, objectives, and methodology are described elsewhere [18]. In brief, 512,713 participants aged 30 to 79 from 10 diverse areas of China were recruited between June 2004 and July 2008 (overall response rate 28%). The study population, identified from public registry records, was not designed to be representative of China as a whole but cover a wide variation in risk factors and diseases. At local assessment centres participants underwent a laptop-based interview providing information on socio-demographic characteristics, aspects of lifestyle (smoking, alcohol intake, diet, physical activity), exposure to passive smoking and household air pollution, and medical history. Trained health professionals undertook physical examination using standard protocols. The Chinese Center for Disease Control and Prevention and the University of Oxford gave ethics clearance for the CKB study. Written informed consent was obtained from all participants. For this report we included participants in the urban region of Suzhou only (*n* = 53,259), for which detailed air pollution data were available. We excluded one assessment centre and its participants (*n* = 257) located outside the urban area of Suzhou. The included assessment centres are spread across an area of approximately 30 km by 15 km.

### Air pollution exposure

Daily 24 h-averaged measurements of inhalable (PM_10_) and fine (PM_2.5_) particulate matter, SO_2_, NO_2_, CO and O_3_ were obtained from 10 fixed-site monitors situated in Suzhou for years 2013 to 2015. We also obtained daily meteorological variables (ground temperature, total precipitation, wind speed, and relative humidity) from five local weather stations. Geographic covariates of elevation, distance to nearest major road, distance to nearest motorway, total length of major roads and motorways in a 1 km radius, and land use (urban or non-urban) were also obtained.

Predictions for pollutant levels at assessment centre locations for each month from January 2013 to December 2015 were derived using Bayesian models. Details of the modelling methodology can be found elsewhere [19]. A two-stage approach was used, applying spatio–temporal models for the meteorological variables then using these predictions (in addition to the five geographic covariates) in spatio–temporal models for each pollutant. Bayesian inference was via integrated nested Laplace approximation (INLA) and the SPDE approach, using the R-INLA package for R software [20]. In these models, predicted values for a random sample of fifty observations showed high correlation with observed values (*r* = 0.80 for CO and 0.87–0.98 for other pollutants).

Monthly pollutant levels were predicted for 77 baseline assessment centres matched to 53,002 participants, each living within 1 km of their respective centre at baseline. The two long-term exposures used in analyses were annual exposure (mean pollutant levels in calendar years 2013, 2014, and 2015) and cumulative exposure (mean pollutant levels from January 2013 to a given month).

### Follow-up for morbidity and mortality

Deaths were identified by electronic linkage to local mortality records, supplemented by annual confirmation of survival through street committees or village administrators and standardised verbal autopsies for mortality without medical attention before death (< 5%). Non-fatal events and any episodes of hospitalisation were captured by disease registers (for cancer, ischaemic heart disease, stroke, and diabetes) and health insurance records. The underlying causes of death and hospital diagnoses were coded in accordance with the International Classification of Diseases, 10th Revision (ICD-10). By 31^st^ December 2015, 1,467 participants (2.8%) had died, and 15 (< 0.1%) were lost to follow-up. The primary outcomes in this analysis were the first occurrence of either non-fatal or fatal cardiovascular (ICD-10: I00-I99) and respiratory (J00-J99) disease from 1^st^ January 2013 to 31^st^ December 2015 when AAP data are available. We also examined specific cardiovascular and respiratory diseases, including ischemic heart disease (IHD) (I20-I25), all-type stroke (I60-I61, I63-I64, I69.0, I69.1, I69.3, I69.4), ischemic stroke (I63), intracerebral haemorrhage (I61), chronic obstructive pulmonary disease (COPD) (J41-J44), and pneumonia (J12-J18). Endpoints capturing other cardiovascular diseases (I00-I16, I27-I52, I62, I65-I89, I95-I99), and other respiratory diseases (J00-J06, J20-J22, J30-J40, J80-J86, J90-J96, J98-J99) were also included (details can be found in eTable 1).

### Statistical analysis

We excluded all participants censored prior to 2013 (*n* = 1482) in addition to participants with the respective cardio-respiratory endpoint(s) of interest recorded prior to 1^st^ January 2013. We restricted our study population to participants with no history of cancer at baseline or incident cancer prior to the start of follow-up. In analyses of all cardiovascular disease and other cardiovascular diseases, participants with history of stroke/transient ischemic attack (TIA) or IHD (*n* = 907) at baseline were excluded. In analyses of stroke, participants with history of stroke/TIA (*n* = 402) were excluded, and in analyses of IHD participants with history of IHD (*n* = 522) were excluded. In analyses of respiratory disease, participants with history of asthma, tuberculosis, or emphysema/bronchitis at baseline (*n* = 2,955) were excluded. Baseline characteristics of the study population and annual levels of individual air pollutants were summarised by means and standard deviations (SDs) or proportions.

Cox proportional hazard models were used to estimate adjusted hazard ratios (HRs) and 95% confidence intervals (CIs) for disease incidence per 10 µg/m^3^ increase in pollutant exposures, or per 100 µg/m^3^ increase for CO exposure. Pollutant exposure was included as a time-varying covariate. In all models time from start of study period was used as the time scale, and models were stratified for baseline age group (5-year groups) and sex, and adjusted for active smoking (never, occasional, ex-, or current smoker), exposure to passive smoking (never, previously, or currently lives with smoker), self-rated health status (excellent, good, fair, or poor), body mass index (kg/m^2^), physical activity level (metabolic equivalent of task per days), alcohol consumption (never, ex-, occasional, monthly, reduced, or weekly drinker), highest education level (no formal schooling, primary school, middle school, high school, college, or university), solid-fuel use for cooking (always clean fuels, switched from solid to clean fuels, always solid fuels, never cooked regularly, other), ambient mean temperature, and prior cardiovascular/respiratory disease (incident respiratory disease prior to the cardiovascular endpoint, or incident cardiovascular disease prior to a respiratory endpoint). Highest education level was chosen as it has been shown to be the best available measure for socioeconomic status in the CKB study. Temperature was included as a time-varying covariate on the same time scale as the pollutant exposure. Additional potential confounders hypothesised a priori to confound the relationship between AAP exposure and cardio-respiratory disease incidence were included. For cardiovascular diseases, these were consumption of fresh fruit and preserved vegetables, current use of hypertensive medication, and systolic blood pressure (SBP) at baseline. For respiratory diseases, these were consumption of fresh fruit, and preserved vegetables respectively, and current use of diabetes medication. We also used a robust variance estimator to account for spatial correlation of participant characteristics and disease incidence within assessment centres. For cardiovascular disease and respiratory disease, further analyses included adjustment variables sequentially and additionally adjusted for household income (6 groups).

We carried out subgroup analyses for pollutant exposures significantly associated with all cardiovascular disease or all respiratory disease, and tested for heterogeneity (or trend, if appropriate) of associations using annual exposures. Additionally, for these pollutant exposures we fit models adjusting for other pollutant exposures. To assess linearity of the associations between these pollutants and cardiovascular and respiratory diseases, we also fit models using natural cubic splines (with 4 degrees of freedom) for annual pollutant exposure.

To ensure the reliability of estimates of associations between pollutant exposures and cardio-respiratory disease, we also estimated the associations with certain infectious and parasitic disease incidence as a “negative control”. This encompassed a limited range of diseases (eTable 1) unlikely to be linked to ambient air pollution exposure but only to potential confounders (e.g. socioeconomic status).

## Results

**Fig. 1:**
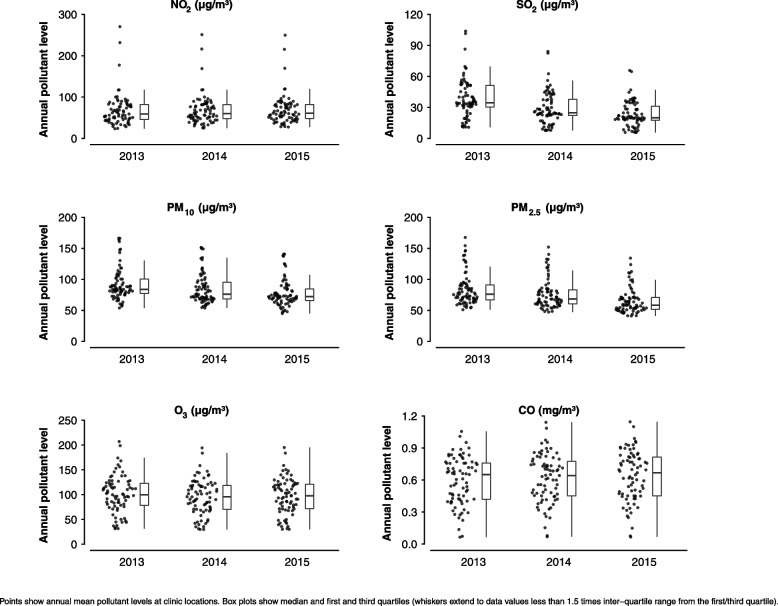
Annual pollutant concentrations by assessment centre location and pollutant, Suzhou (2013–2015)

Mean pollutant concentrations and long-term trends through the study period (2013–2015) varied considerably between pollutants (Table 1, Fig. 1 and eFigure 1). Mean levels of particulate matter declined 15.5% and 21.9% between 2013 and 2015 for PM_10_ and PM_2.5,_ respectively. Gaseous pollutant concentrations showed less consistent long-term trends and concentrations varied substantially between assessment centres. Mean levels of SO_2_ fell by 39.9% between 2013 and 2015. Annual levels of PM_2.5_ were strongly positively correlated with PM_10_ and NO_2_, while annual O_3_ levels were negatively correlated with PM_10_ and PM_2.5_ (eFigure 2).

**Table 1:** Summary of predicted annual pollutant levels at assessment centre locations

	**2013**					**2014**					**2015**					
**Pollutant (μg/m**^**3**^**)**	**Mean**	**SD**	**P5**	**P95**	**IQR**	**Mean**	**SD**	**P5**	**P95**	**IQR**	**Mean**	**SD**	**P5**	**P95**	**IQR**	**% change of mean from 2013 to 2015**
NO_2_	68.4	39.5	30.9	117.3	36.5	68.2	36.7	32.3	117.5	33.7	69.4	36.1	33.8	119.4	33.8	+ 1.4
SO_2_	38.6	18.9	11.7	68.4	20.9	29.4	15.5	8.4	52.5	15.5	23.2	12.3	6.3	40.3	13.7	-39.9
PM_10_	91.9	25.8	60.2	151.0	23.3	85.2	24.4	58.2	137.7	26.9	77.6	21.9	50.0	127.5	18.9	-15.5
PM_2.5_	83.6	25.8	56.5	140.5	24.1	76.3	23.4	51.3	128.5	22.4	65.3	19.9	44.9	110.2	19.0	-21.9
O_3_	100.9	37.8	37.5	161.8	44.3	95.1	35.6	35.5	145.6	48.3	96.8	36.0	35.9	149.0	49.4	-4.1
CO (mg/m^3^)	0.59	0.22	0.24	0.89	0.34	0.62	0.23	0.26	0.95	0.32	0.64	0.24	0.26	0.96	0.36	+ 8.0

Summary statistics calculated across annual predicted pollutant levels at all assessment centre locations

*SD* Standard deviation, *P5* 5th Percentile. *P95* 95th Percentile, *IQR* Inter-quartile range

Baseline characteristics for men and women included in analyses are presented in Table 2. The mean age of participants was 51.9 years for men and 51.5 years for women. While current regular smoking was highly prevalent (68.7%) among men, it was rare in women (0.4%). Similarly, regular alcohol consumption was common (41.1%) in men but not in women (0.6%). Self-rated health was slightly better among men (33.4% excellent and 7.1% poor) than women (26.6% excellent and 11.9% poor).

**Table 2:** Baseline characteristics of participants in Suzhou

	**Men** **(*n* = 20,846)**	**Women** **(*n* = 29,561)**
**Total follow-up time (CVD), person-years**	55,962	80,193
**Total follow-up time (respiratory disease), person-years**	57,706	82,830
**Age, years, mean (SD)**	51.9 (10.1)	51.5 (10.2)
**Education**		
No formal school	9.7	43.3
Primary school	40.0	26.8
Middle school	36.4	22.8
High school and above	13.8	7.1
**Smoking**		
Never smoker	7.7	99.1
Occasional smoker	10.8	0.5
Ex regular smoker	12.7	0.0
Current regular smoker	68.7	0.4
**Passive smoking**		
Never	22.9	7.5
Yes, but not now	57.1	27.5
Yes, at present	20	65
**Alcohol consumption**		
Never	16.2	89.1
Ex-regular / Reduced intake	10.6	0.3
Occasional / Monthly	32.2	10.0
Current regular	41.1	0.6
**Self-rated health**		
Excellent	33.4	26.6
Good	32.9	31.7
Fair	26.5	29.8
Poor	7.1	11.9
**Physical activity, MET-hr/day, mean (SD)**	27.8 (15.9)	24.4 (14.3)
**Self-reported disease**		
Hypertension	10.8	11.5
Stroke/TIA	1.1	0.6
TB	1.6	0.6
CHD	1	1
Asthma	0.7	0.8
Emphysema/Bronchitis	4.5	4.5
**Family history of disease**^**a**^		
Stroke	18.7	17.0
Myocardial infarction	1.9	2.1
**Cardiorespiratory symptoms**^**b**^		
Breathlessness	2.0	3.8
Chest pain	1.0	2.2
Chronic cough	4.8	2.3

Values are percentages, unless otherwise stated

^a^ Family history defined as baseline reported disease prevalence in mother and/or father

^b^ Breathlessness and chest pain defined as being “short of breath”, and “slowing down due to chest discomfort”, respectively, when “walking on level ground with health people of the same age”. Chronic cough defined as “coughing frequently for ≥ 3 months"

During the study period (including a total of 135,199 person-years of follow-up for CVD and 130,917 person-years for respiratory disease), 2,563 participants had a fatal or non-fatal cardiovascular event, and 1,764 had a respiratory disease event. Rates of both first cardiovascular event and first respiratory disease event were slightly higher in men (20.0 cardiovascular and 14.5 respiratory events per 1,000 person-years) compared to women (18.6 cardiovascular and 12.8 respiratory events per 1,000 person-years).

**Fig. 2:**
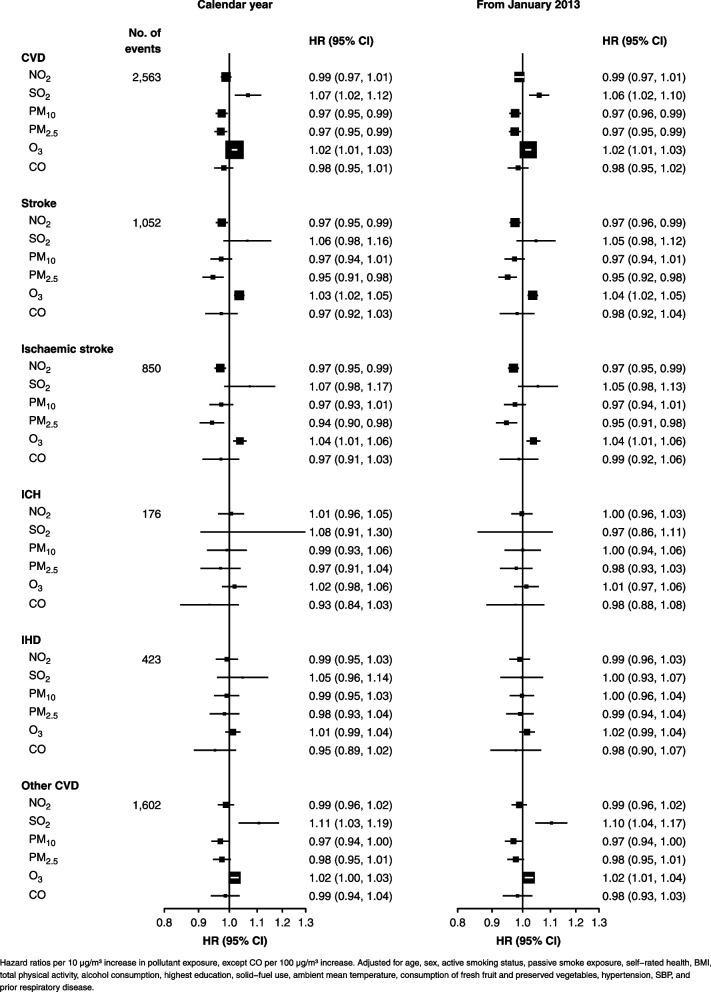
Associations between cardiovascular diseases and long-term pollutant exposures

Adjusted HRs per 10 µg increase in long-term pollutant exposures (per 100 µg for CO) for cardiovascular diseases are shown in Fig. 2. Increased SO_2_ exposure is significantly associated with all cardiovascular disease (HR (95%CI): 1.07 (1.02, 1.12)) and other cardiovascular disease (1.11 (1.03, 1.19)). O_3_ exposure is moderately positively associated with all cardiovascular disease, all-type stroke, and ischemic stroke (1.02 (1.01, 1.03), 1.03 (1.02, 1.05), and 1.04 (1.01, 1.06)). PM_10_ and PM_2.5_ are moderately inversely associated with all cardiovascular disease (0.97 (0.95, 0.99)), in particular ischaemic stroke for PM_2.5_, and similarly NO_2_ with ischaemic stroke (0.97 (0.95, 0.99)).

**Fig. 3:**
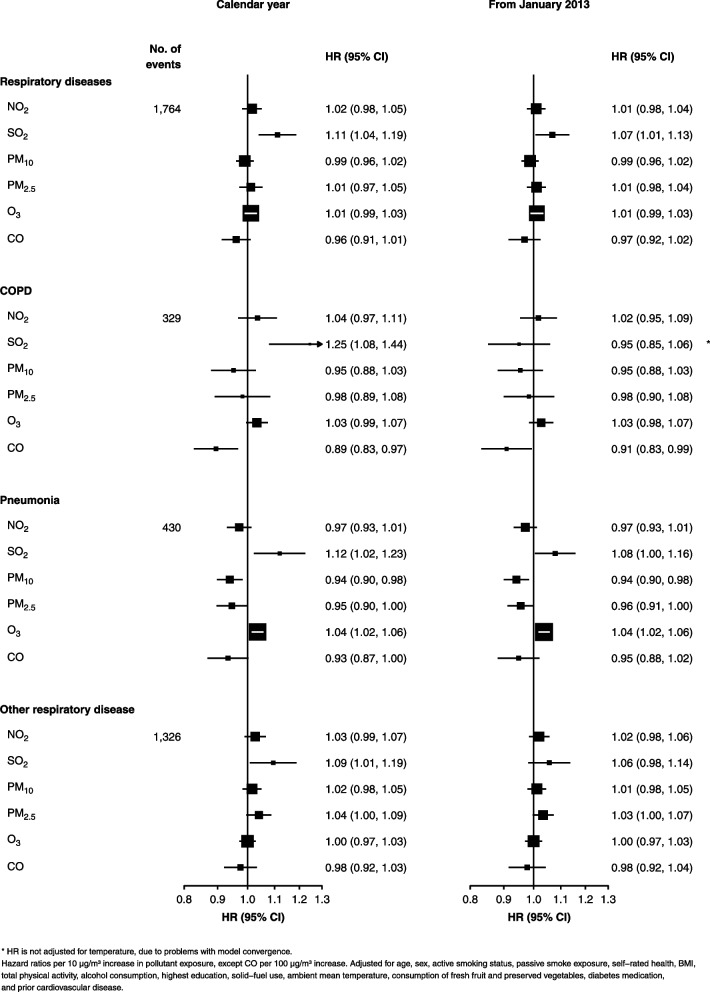
Associations between respiratory diseases and long-term pollutant exposures

Adjusted HRs for respiratory diseases are shown in Fig. 3. Increased SO_2_ exposure is significantly associated with all respiratory disease (1.11 (1.04, 1.19)), COPD (1.25 (1.08, 1.44)), pneumonia (1.12 (1.02, 1.23)), and other respiratory disease (1.09 (1.01, 1.19)). O_3_ exposure is associated with increased risk of pneumonia (1.04 (1.02, 1.06)). PM_10_ and PM_2.5_ are inversely associated with pneumonia (0.94 (0.90, 0.98) and 0.95 (0.90, 1.00), respectively), and CO is inversely associated with COPD (0.89 (0.83, 0.97)).

Analyses using cumulative pollutant exposures from January 2013 show similar results to analyses using annual exposures. Analyses of all cardiovascular disease and all respiratory disease with sequential adjustment for potential confounders show minimal change in estimates after adjustment for age, sex, and selected lifestyle and environmental factors (eFigure 8).

After adjustment for NO_2_ or CO, SO_2_ exposure remains significantly associated with cardiovascular disease, however adjustment for PM_2.5_, PM_10_ or O_3_ attenuates the association (eFigure 3). Adjustment for other pollutants does not attenuate the associations between O_3_ exposure and all-type stroke and ischemic stroke. After adjustment for other pollutants, SO_2_ exposure is significantly associated with respiratory disease, but not pneumonia (eFigure 4).

Results of stratified analyses for SO_2_ and O_3_ are presented in eTables 2 and 3. Heterogeneous associations are observed for SO_2_ and cardiovascular disease between different exposures to passive smoking (*p* = 0.04), and for O_3_ and respiratory disease between different smoking (*p* < 0.01).

Associations between SO_2_ and O_3_ and cardiovascular diseases are not significantly non-linear (*p* = 0.45 and *p* = 0.46, respectively). However, associations with respiratory diseases show significantly non-linearity (*p* = 0.002 and *p* < 0.001), with approximately linear associations above 30 µg/m^3^ SO_2_ and above 100 µg/m^3^ O_3_ as shown in eFigure 5.

Increased SO_2_ exposure is also associated with a higher risk of all-type and ischemic stroke when using monthly pollutant exposures (eFigure 6). However, the associations with all cardiovascular disease and other cardiovascular disease are not significant, as seen when using annual SO_2_ exposure. Monthly O_3_ is also associated with cardiovascular disease, all-type stroke, and ischemic stroke. For respiratory diseases, monthly SO_2_ exposure remains associated only with increased risk of pneumonia (eFigure 7).

Associations between annual and monthly pollutant exposures and infectious and parasitic disease incidence (467 events) are not significant (eTable 4).

## Discussion

In this prospective cohort study in the urban region Suzhou in China we found some evidence for associations between long-term exposure to ambient SO_2_ and O_3_ pollution and increased risk of cardio-respiratory events. In particular, higher SO_2_ exposure is associated with increased risk of COPD and pneumonia incidence, and higher O_3_ with increased risk of ischaemic stroke.

Most existing studies focus on short-term SO_2_ exposure, and the associations of long-term exposure with cardio-respiratory health remain poorly understood [21]. Several previous cohort studies in China have reported positive associations between SO_2_ exposure and cardio-respiratory mortality, but effect sizes tended to be smaller than that observed in the present study [9–11]. This may be explained by relatively crude exposure assessment methods (e.g. fixed-site monitoring data assigned to residential zip codes) and lack of detailed adjustment for confounders in previous studies. We found some evidence of a threshold effect such that the harm of SO_2_ may exceed individuals’ ability to cope at certain levels, but this needs to be verified in other large cohort studies.

SO_2_ is often hypothesized as a proxy for other combustion-based pollutants [22] such as PM_2.5_, instead of having direct long-term health impact on major cardio-respiratory outcomes. We observed a lower correlation between PM_2.5_ and SO_2_ than seen in other studies, both in China [10] and elsewhere [23]. This could be due to our model of exposure assessment accounting for a greater degree of small-scale spatial and temporal variation, potentially reducing correlations between pollutants seen when using averages from fixed-site monitors [24]. It may also reflect increasingly stringent SO_2_ emission control (e.g., flue-gas desulphurisation) and the rapidly expanding car fleet in China in recent years, shifting relative PM contribution of older coal-fired power plants and industries to less sulphur-intense sources [25]. We found SO_2_ associated with respiratory disease after adjustment for other pollutants, suggesting some amount of an independent effect rather than SO_2_ being solely representative of exposure to other pollutants as previously hypothesised [22]. However, adjustment for some other pollutants did attenuate the association between SO_2_ exposure and cardiovascular disease.

To our knowledge, no study has examined the long-term cardio-respiratory effects of O_3_ exposure in China, and findings are mixed from the few studies performed in high-income countries. While some have found O_3_ exposure associated with cardio-respiratory mortality [26, 27] even after adjustment for PM_2.5_ [28, 29], others have found no clear association [30, 31]. We found modest associations between O_3_ exposure and cardiovascular disease (primarily ischemic stroke), including when adjusting for exposure to other pollutants. This provides new prospective study evidence, supporting previously observed associations in several large cohort studies [32]. The discrepancy between the present and some previous studies may be attributed to the significantly higher O_3_ exposure recorded in this study (mean 86.9 µg/m^3^ versus ~ 50–55 µg/m^3^) [30] and more extensive adjustment for individual-level confounders than in previous studies. Further studies to investigate the apparent non-linear dose–response relationship between O_3_ and respiratory disease is warranted.

The exact mechanisms linking O_3_ and SO_2_ with cardio-respiratory diseases remain to be confirmed. For example, experimental studies have shown O_3_ to be associated with numerous major pathways of cardiovascular disease development including oxidative stress, and inflammatory pathways [33, 34]. Similarly, experimental and toxicological studies have shown SO_2_ exposure capable of inducing oxidative damage and mitochondrial dysfunction in the heart and lungs of mice [35, 36] and associated with reduced cardiac vagal control in humans [37].

While this study found moderate inverse associations between long-term particulate matter exposure and cardio-respiratory disease, most previous studies observed significant positive associations, including the few existing cohort studies in China [9, 17, 38]. A large nationwide cohort study in China reported overall positive associations between PM_2.5_ and cardiovascular disease incidence and mortality, but found no clear association below 70 µg/m^3^ or in urban areas [17]. These results may reflect substantial regional variation in emission sources, and thus chemical composition, of particulate matter, with solid fuels and less efficient coal-fired power plants dominating rural AAP [39], which may be more harmful than particulate matter from mobile vehicles or industries [40], two major contributors of urban AAP. Declining PM levels may also have resulted in observable benefits on cardio-respiratory disease, as previously documented for mortality [41] and subclinical markers of inflammation [42] in China. It may also be due to residual confounding, especially by other pollutants, or the time delay between exposure and development of disease.

An important strength of this study is the inclusion of both non-fatal and fatal incident disease. A considerable proportion of the long-term burden relating to AAP exposure is likely captured by morbidity only, possibly more sensitive to AAP exposure than mortality [43, 44]. In addition, we used a spatio–temporal model to assign air pollution exposure instead of averages from fixed-site monitors, to attempt to account for small-scale variability in exposure and give a more robust exposure assessment [43]. Further, we included adjustment for a ranged of individual-level covariates and analysed all six criteria ambient air pollutants.

This study is limited by the short follow-up time coupled with latency in the development of numerous cardio-respiratory diseases. It is unlikely to capture all participants’ historic AAP exposure that may be associated with disease development. We attempted to alleviate this by examining disease endpoints representative of potentially shorter latency. For disease incidence not captured within the 3-year exposure period, extrapolation of AAP concentrations to additional years of follow-up was possible, as seen in other studies [16, 44]. However, given the substantial long-term trends seen in AAP, extrapolation would be unlikely to capture the exposure accurately [45]. We were unable to assign exposure based on residential addresses (though the CKB sampling strategy ensures that the vast majority of participants lived within 1 km of their assessment centre) and there is inherent measurement error in exposures predicted from a modelling strategy. Despite our adjustment for potential confounders at the individual level, residual confounding from factors such as socioeconomic status and health-seeking behaviour remain plausible.

## Conclusion

The findings of this study provide new evidence that long-term exposure to O_3_ and SO_2_ is associated with increased risk of both cardiovascular and respiratory diseases. In contrast to the continuous declining trend of PM_2.5_ in China, anthropogenic O_3_ has been increasing rapidly in the past decade, prompting growing concerns of its potentially increasing proportional public health impact [46]. Our findings support more stringent regulatory policy to control other key criteria pollutants, in addition to PM_2.5_. Further cohort studies with greater geographic coverage, larger sample size, and longer follow-up will help to clarify the magnitude of associations between AAP exposures and disease.

## Supplementary Information

**Additional file 1.**

## Data Availability

The China Kadoorie Biobank (CKB) is a global resource for the investigation of lifestyle, environmental, blood biochemical and genetic factors as determinants of common diseases. The CKB study group is committed to making the cohort data available to the scientific community in China, the UK and worldwide to advance knowledge about the causes, prevention and treatment of disease. For detailed information on what data is currently available to open access users and how to apply for it, visit: http://www.ckbiobank.org/site/Data+Access. Researchers who are interested in obtaining the raw data from the China Kadoorie Biobank study that underlines this paper should contact ckbaccess@ndph.ox.ac.uk. A research proposal will be requested to ensure that any analysis is performed by bona fide researchers and—where data is not currently available to open access researchers—is restricted to the topic covered in this paper.

## Abbreviations

AAP: Ambient air pollution
CKB: China Kadoorie Biobank
INLA: Integrated nested Laplace approximation
SPDE: Stochastic partial differential equation
ICD-10: International Classification of Diseases, 10th Revision
IHD: Ischemic heart disease
COPD: Chronic obstructive pulmonary disease
TIA: Transient ischemic attack
HR: Hazard ratio
CI: Confidence intervals
SBP: Systolic blood pressure
