# Supplementary material for: Long-term ambient air pollution exposure and cardio-respiratory disease in China: findings from a prospective cohort study
Source: Environ Health. 2023 Mar 27;22:30. doi: 10.1186/s12940-023-00978-9 (PMC10041804; doi:10.1186/s12940-023-00978-9)
Supplement: Supplementary file 1 — Additional file 1. [file 12940_2023_978_MOESM1_ESM.docx]

Long-term ambient air pollution exposure and cardio-respiratory disease in China: Findings from a prospective cohort study

Neil Wright, Katherine Newell, Ka Hung Chan, Simon Gilbert, Alex Hacker, Yan Lu, Yu Guo, Pei Pei, Canqing Yu, Jun Lv, Junshi Chen, Liming Li, Om Kurmi, Zhengming Chen, Kin Bong Hubert Lam, Christiana Kartsonaki

Table of Content

# eTable 1: ICD-10 codes contained within cardiovascular and respiratory endpoints classed as “other”, and the infectious and parasitic disease endpoint

| **Endpoint** | **ICD-10 codes and descriptions** | **Number (%) of endpoints during follow-up*** |
| --- | --- | --- |
| Other respiratory diseases (n=1323) | Bronchitis, not specified as acute or chronic (J40) | 440 (33.3%) |
|  | Other respiratory diseases including; diseases of bronchus, not elsewhere classified (J98.0), pulmonary collapse (J98.1), interstitial emphysema (J98.2), compensatory emphysema (J98.3); other disorders of lung (J98.4), diseases of mediastinum not elsewhere classified (J98.5), disorders of diaphragm (J98.6), other specified respiratory disorders (J98.8), respiratory disorder, unspecified (J98.9) | 575 (43.5%) |
|  | Other upper respiratory diseases including; vasomotor and allergic rhinitis (J30), chronic rhinitis, nasopharyngitis and pharyngitis (J31), chronic sinusitis (J32), nasal polyp (J33), other and unspecified disorders of nose and nasal sinuses (J34), chronic diseases of tonsils and adenoids (J35), peritonsillar abscess (J36), chronic laryngitis and laryngotracheitis (J37), disease of vocal cords and larynx, not elsewhere classified (J38) and others (J39) | 141 (10.7%) |
|  | All others including upper respiratory infection (J00-J06), other lower respiratory infection (J20-J22), other respiratory diseases principally affecting the interstitium (J80-J84), suppurative and necrotic conditions of the lower respiratory tract (J85-J86), other diseases of the pleura (J90-J94), intraoperative and post-operative complications of respiratory system (J95), respiratory failure not elsewhere classified (J96) respiratory disorders in diseases classified elsewhere (J99) | 167 (12.6%) |
| Other cardiovascular diseases (n=1601) | Diseases of veins, lymphatic vessels and lymph nodes, not elsewhere classified except "other non-infective disorders of lymphatic vessels and lymph nodes (I80-I88) | 637 (39.8%) |
|  | Hypertensive diseases (I10-I16 ) | 682 (42.6%) |
|  | Heart failure, complications and ill-defined descriptions of heart disease, and other heart disorders in diseases classified elsewhere (I50-I52) | 143 (8.9%) |
|  | All others including acute rheumatic fever (I00-I02), chronic rheumatic heart diseases (I05-I09), pulmonary heart disease and diseases of pulmonary circulation (I26-I28), other forms of heart disease (I30-I50), diseases of arteries, arterioles and capillaries (I70-I79),other and unspecified disorders of the circulatory system (I95-I99) | 139 (8.7%) |
| Certain infectious and parasitic diseases (n=486) | Unspecified infectious disease (B99) | 192 (39.5%) |
|  | Infectious gastroenteritis and colitis, unspecified (A09) | 73 (15.0%) |
|  | Tuberculosis (A15-A19) | 50 (10.3%) |
|  | Viral infections characterized by skin and mucous membrane lesions (B00-B09) | 61 (12.6%) |
|  | Other bacterial intestinal infections (A04) | 29 (6.0%) |
|  | Other bacterial diseases (A30-A49) | 27 (5.6%) |
|  | Others including, Helminthiases (B65-B83), viral hepatitis (B15-B19), mycoses (B35-B36), sequelae of infectious and parasitic diseases (B90-B94), other viral diseases (B25-B34), human immunodeficiency virus [HIV] disease (B20-B24) | 54 (11.1%) |

# eTable 2: Associations between sulphur dioxide exposure and cardiovascular and respiratory diseases, by subgroups

|  | **CVD** | | **Respiratory disease** | |
| --- | --- | --- | --- | --- |
|  | **HR (95% CI)** | **P-value for heterogeneity** | **HR (95% CI)** | **P-value for heterogeneity** |
| **Sex** |  |  |  |  |
| Men | 1.07 (1.03, 1.11) | 0.82 | 1.15 (1.06, 1.24) | 0.26 |
| Women | 1.08 (1.01, 1.15) |  | 1.07 (0.99, 1.17) |  |
| **Age at baseline, years** |  |  |  |  |
| <60 | 1.07 (0.98, 1.16) | 0.89 | 1.10 (1.00, 1.21) | 0.79 |
| 60-69 | 1.06 (0.99, 1.14) |  | 1.19 (1.10, 1.29) |  |
| 70+ | 1.08 (0.98, 1.20) |  | 1.01 (0.87, 1.18) |  |
| **Education** |  |  |  |  |
| No formal education | 1.10 (1.04, 1.16) | 0.25 | 1.10 (1.01, 1.19) | 0.21 |
| Primary school | 1.04 (0.98, 1.11) |  | 1.13 (1.02, 1.25) |  |
| Middle School | 1.01 (0.92, 1.11) |  | 1.14 (0.99, 1.31) |  |
| High school or above | 1.13 (0.93, 1.38) |  | 1.27 (1.05, 1.55) |  |
| **Smoking** |  |  |  |  |
| Never smoker | 1.07 (1.01, 1.15) | 0.33 | 1.08 (0.99, 1.16) | 0.38 |
| Occasional smoker | 1.28 (1.04, 1.59) |  | 1.01 (0.77, 1.33) |  |
| Ex regular smoker | 1.03 (0.91, 1.17) |  | 1.23 (1.03, 1.47) |  |
| Smoker | 1.04 (0.97, 1.13) |  | 1.16 (1.06, 1.27) |  |
| **Ever lived with smoker** |  |  |  |  |
| Never | 1.21 (1.08, 1.36) | 0.04 | 1.12 (0.99, 1.28) | 0.54 |
| Yes, but not now | 1.02 (0.96, 1.09) |  | 1.07 (0.99, 1.16) |  |
| Yes, at present | 1.06 (1.00, 1.13) |  | 1.14 (1.05, 1.25) |  |
| **Solid fuel use** |  |  |  |  |
| Always clean fuels | 1.15 (1.03, 1.29) | 0.45 | 1.29 (1.11, 1.49) | 0.24 |
| Switched from solid to clean fuels | 1.07 (0.99, 1.17) |  | 1.08 (0.98, 1.19) |  |
| Always solid fuels | 1.09 (1.01, 1.17) |  | 1.09 (0.98, 1.21) |  |
| Never cooked regularly | 1.00 (0.91, 1.11) |  | 1.21 (1.07, 1.37) |  |
| Other | 1.14 (0.94, 1.38) |  | 1.13 (0.96, 1.34) |  |
| **BMI, kg/m²** |  |  |  |  |
| <18.5 | 0.99 (0.79, 1.23) | 0.15 | 1.16 (0.91, 1.47) | 0.98 |
| 18.5-24.9 | 1.04 (0.98, 1.11) |  | 1.10 (1.03, 1.19) |  |
| 25+ | 1.10 (1.03, 1.18) |  | 1.12 (1.02, 1.24) |  |
| **Self-rated health** |  |  |  |  |
| Excellent | 1.05 (0.96, 1.14) | 0.54 | 1.19 (1.09, 1.31) | 0.16 |
| Good | 1.09 (1.01, 1.17) |  | 1.15 (1.05, 1.25) |  |
| Fair | 1.02 (0.93, 1.11) |  | 1.06 (0.96, 1.17) |  |
| Poor | 1.12 (0.98, 1.29) |  | 1.00 (0.86, 1.17) |  |
| **Prior respiratory disease** |  |  |  |  |
| No | 1.06 (1.01, 1.12) | 0.98 |  |  |
| Yes | 1.06 (0.91, 1.24) |  |  |  |
| **Prior CVD** |  |  |  |  |
| No |  |  | 1.11 (1.03, 1.19) | 0.96 |
| Yes |  |  | 1.11 (0.96, 1.27) |  |

Hazard ratios per 10 µg/m³ increase in pollutant exposure, except CO per 100 µg/m³ increase. All analyses adjusted for age, sex, active smoking status, passive smoke exposure, self-rated health, BMI, total physical activity, alcohol consumption, highest education, solid-fuel use, ambient mean temperature, consumption of fresh fruit and preserved vegetables. Analyses of cardiovascular diseases also adjusted for hypertension, SBP, and prior respiratory disease. Analyses of respiratory diseases also adjusted for diabetes medication and prior cardiovascular disease.

# eTable 3: Associations between ozone exposure and cardiovascular and respiratory diseases, by subgroups

|  | **CVD** | | **Respiratory disease** | |
| --- | --- | --- | --- | --- |
|  | **HR (95% CI)** | **P-value for heterogeneity** | **HR (95% CI)** | **P-value for heterogeneity** |
| **Sex** |  |  |  |  |
| Men | 1.01 (1.00, 1.03) | 0.71 | 1.02 (0.99, 1.05) | 0.59 |
| Women | 1.02 (1.00, 1.03) |  | 1.01 (0.99, 1.03) |  |
| **Age at baseline, years** |  |  |  |  |
| <60 | 1.02 (1.01, 1.04) | 0.47 | 1.01 (0.98, 1.03) | 0.79 |
| 60-69 | 1.02 (1.00, 1.03) |  | 1.04 (1.02, 1.06) |  |
| 70+ | 1.01 (0.99, 1.04) |  | 0.98 (0.95, 1.02) |  |
| **Education** |  |  |  |  |
| No formal education | 1.03 (1.01, 1.04) | 0.69 | 1.03 (1.01, 1.05) | 0.23 |
| Primary school | 1.00 (0.99, 1.02) |  | 1.00 (0.97, 1.04) |  |
| Middle School | 1.00 (0.97, 1.02) |  | 1.00 (0.96, 1.04) |  |
| High school or above | 1.06 (1.02, 1.10) |  | 1.02 (0.96, 1.07) |  |
| **Smoking** |  |  |  |  |
| Never smoker | 1.02 (1.01, 1.03) | 0.10 | 1.01 (0.99, 1.03) | <0.01 |
| Occasional smoker | 1.05 (1.00, 1.11) |  | 0.89 (0.84, 0.95) |  |
| Ex regular smoker | 1.03 (0.99, 1.06) |  | 1.01 (0.96, 1.06) |  |
| Smoker | 1.00 (0.99, 1.02) |  | 1.03 (1.01, 1.06) |  |
| **Ever lived with smoker** |  |  |  |  |
| Never | 1.04 (1.01, 1.07) | 0.07 | 1.04 (0.99, 1.08) | 0.52 |
| Yes, but not now | 1.00 (0.98, 1.02) |  | 1.01 (0.99, 1.03) |  |
| Yes, at present | 1.02 (1.01, 1.04) |  | 1.00 (0.97, 1.04) |  |
| **Solid fuel use** |  |  |  |  |
| Always clean fuels | 1.02 (0.99, 1.05) | 0.46 | 1.02 (0.96, 1.08) | 0.70 |
| Switched from solid to clean fuels | 1.03 (1.01, 1.04) |  | 1.00 (0.98, 1.03) |  |
| Always solid fuels | 1.01 (0.99, 1.03) |  | 1.02 (0.99, 1.04) |  |
| Never cooked regularly | 1.01 (0.99, 1.04) |  | 1.03 (1.01, 1.06) |  |
| Other | 1.04 (0.99, 1.09) |  | 1.01 (0.95, 1.07) |  |
| **BMI, kg/m²** |  |  |  |  |
| <18.5 | 1.00 (0.94, 1.06) | 0.60 | 0.98 (0.92, 1.05) | 0.29 |
| 18.5-24.9 | 1.02 (1.00, 1.03) |  | 1.01 (0.99, 1.03) |  |
| 25+ | 1.02 (1.01, 1.03) |  | 1.02 (1.00, 1.05) |  |
| **Self-rated health** |  |  |  |  |
| Excellent | 1.03 (1.01, 1.05) | 0.21 | 1.03 (0.99, 1.06) | 0.55 |
| Good | 1.01 (1.00, 1.03) |  | 1.02 (0.99, 1.05) |  |
| Fair | 1.00 (0.98, 1.02) |  | 1.00 (0.97, 1.02) |  |
| Poor | 1.04 (1.01, 1.07) |  | 1.01 (0.97, 1.04) |  |
| **Prior respiratory disease** |  |  |  |  |
| No | 1.02 (1.01, 1.03) | 0.27 |  |  |
| Yes | 1.00 (0.96, 1.04) |  |  |  |
| **Prior CVD** |  |  |  |  |
| No |  |  | 1.01 (0.99, 1.03) | 0.50 |
| Yes |  |  | 1.02 (0.99, 1.06) |  |

Hazard ratios per 10 µg/m³ increase in pollutant exposure, except CO per 100 µg/m³ increase. All analyses adjusted for age, sex, active smoking status, passive smoke exposure, self-rated health, BMI, total physical activity, alcohol consumption, highest education, solid-fuel use, ambient mean temperature, consumption of fresh fruit and preserved vegetables. Analyses of cardiovascular diseases also adjusted for hypertension, SBP, and prior respiratory disease. Analyses of respiratory diseases also adjusted for diabetes medication and prior cardiovascular disease.

# eTable 4: Associations between certain infectious and parasitic diseases and pollutant exposures

|  | **HR (95% CI)** | |
| --- | --- | --- |
| **Pollutant** | **Annual exposure** | **Monthly exposure** |
| NO_2_ | 0.99 (0.95, 1.04) | 0.99 (0.95, 1.03) |
| SO_2_ | 1.06 (0.94, 1.18) | 1.05 (0.95, 1.16) |
| PM_10_ | 0.97 (0.92, 1.02) | 0.98 (0.93, 1.03) |
| PM_2.5_ | 0.99 (0.92, 1.05) | 0.99 (0.92, 1.06) |
| O_3_ | 1.01 (0.99, 1.04) | 1.01 (0.98, 1.03) |
| CO | 1.00 (0.93, 1.07) | 1.00 (0.94, 1.07) |

Hazard ratios per 10 µg/m³ increase in pollutant exposure, except CO per 100 µg/m³ increase. Adjusted for age, sex, active smoking status, passive smoke exposure, self-rated health, BMI,
total physical activity, alcohol consumption, highest education, solid-fuel use, ambient mean temperature, and consumption of fresh fruit and preserved vegetables.

# eFigure 1: Temporal trends in predicted monthly pollutant concentrations by assessment centre location and pollutant, Suzhou (2013-2015)


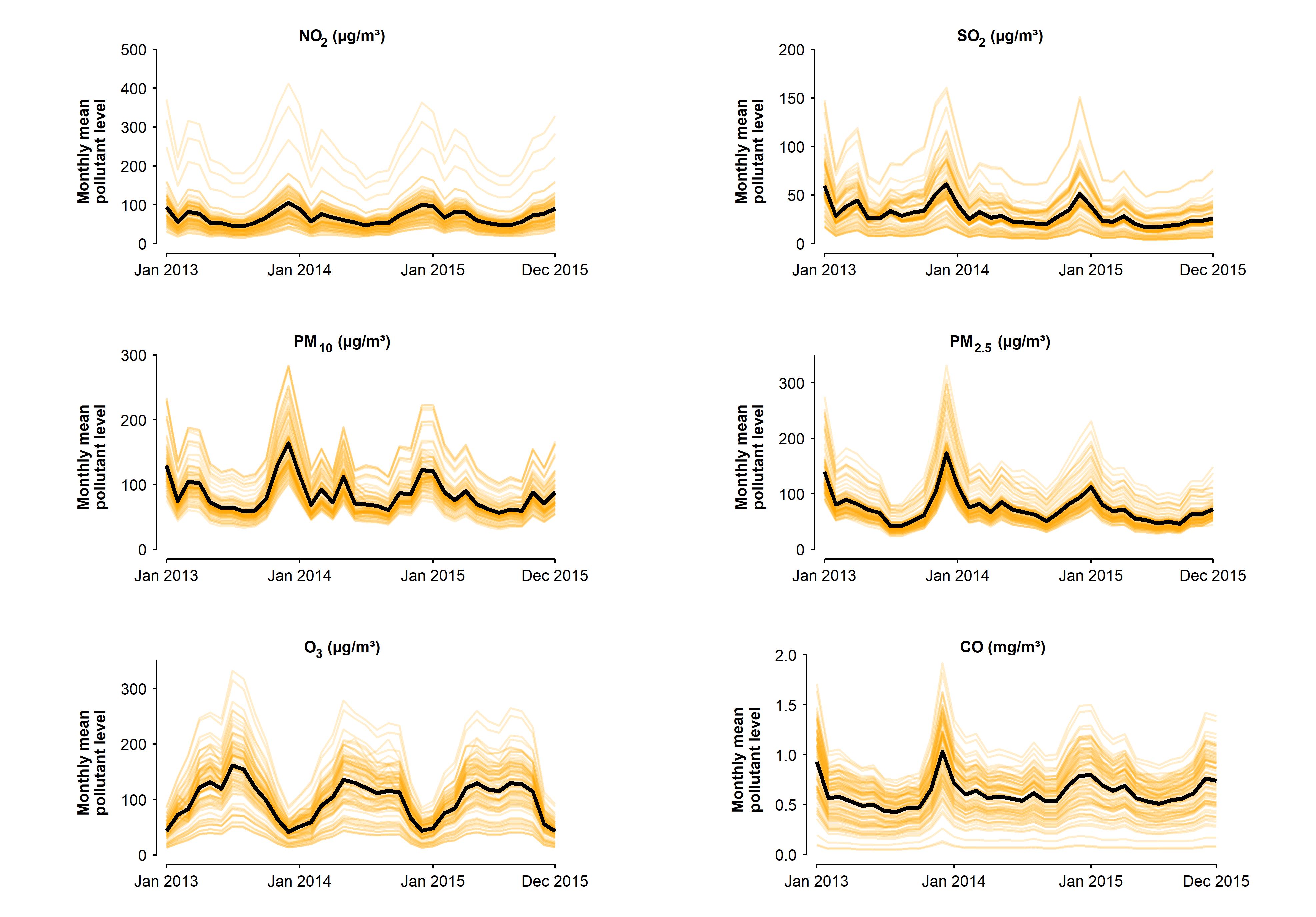


Light orange lines show monthly pollutant levels at assessment centre locations. Black lines are mean monthly pollutant levels across assessment centre locations.

# eFigure 2: Pearson correlation coefficients between annual mean pollutant levels


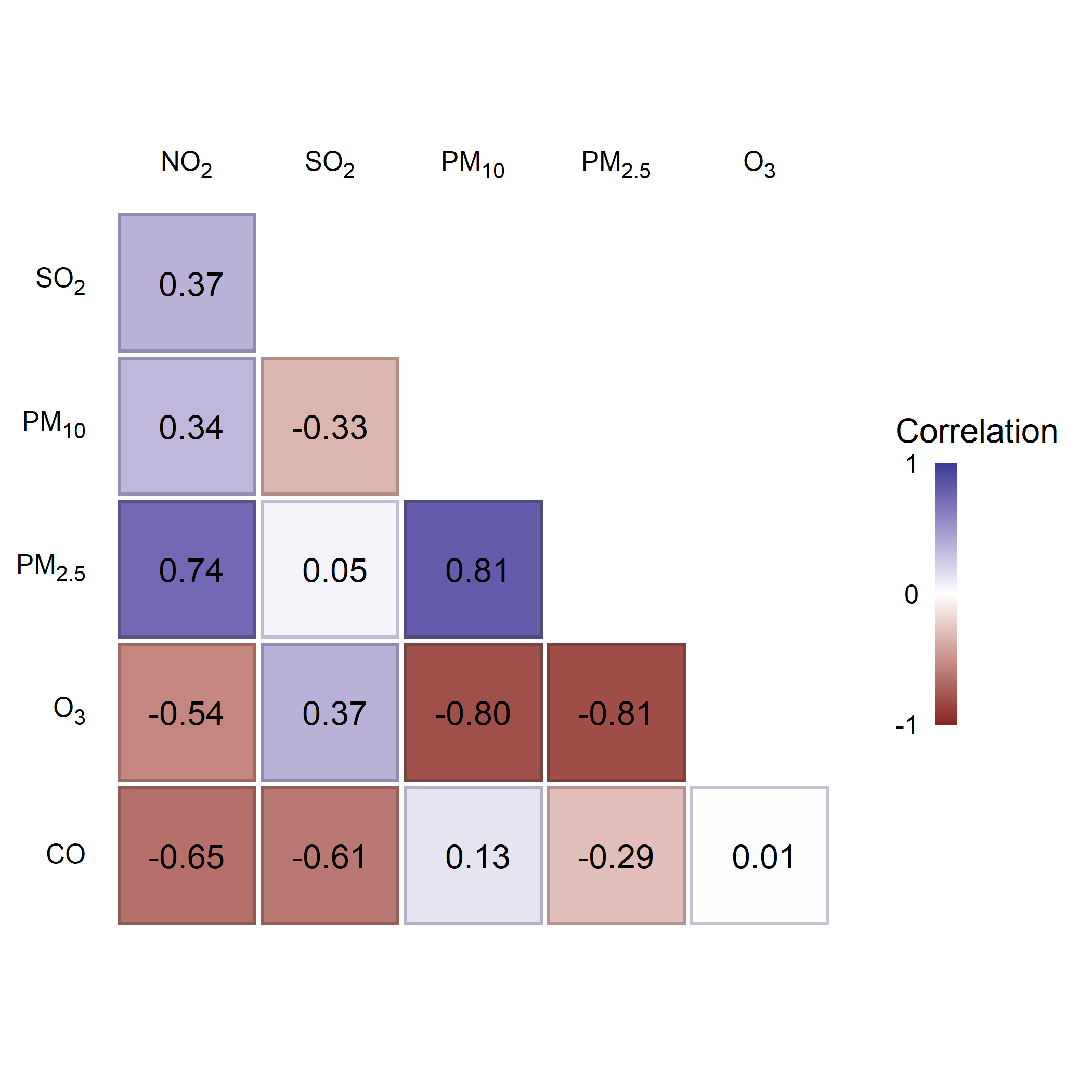


# eFigure 3: Associations between cardiovascular diseases and pollutant exposures, adjusted for other pollutants


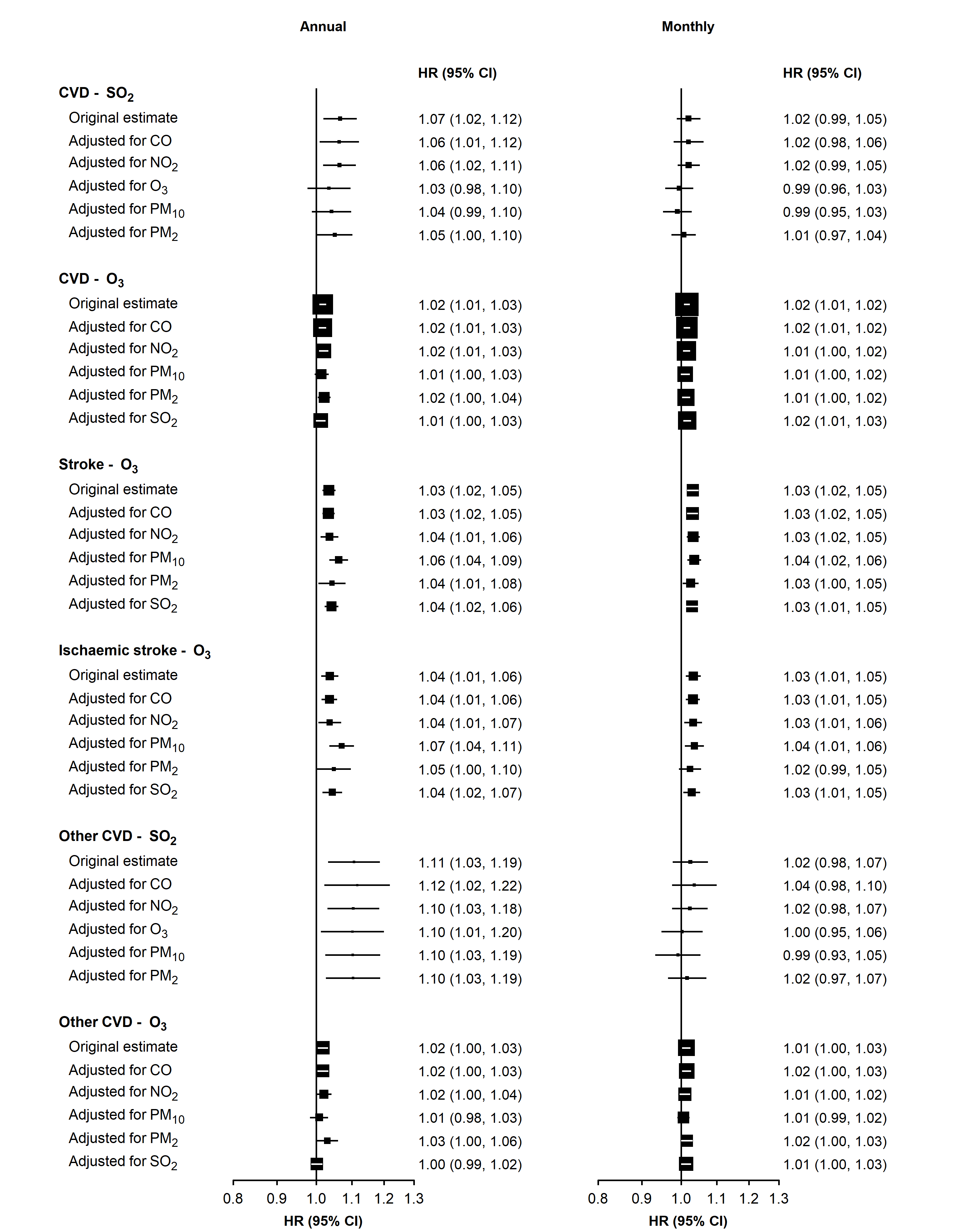


Hazard ratios per 10 µg/m³ increase in pollutant exposure, except CO per 100 µg/m³ increase. Adjusted for age, sex, active smoking status, passive smoke exposure, self-rated health, BMI, total physical activity, alcohol consumption, highest education, solid-fuel use, ambient mean temperature, consumption of fresh fruit and preserved vegetables, hypertension, SBP, and prior respiratory disease.

# eFigure 4: Associations between respiratory diseases and pollutant exposures, adjusted for other pollutants


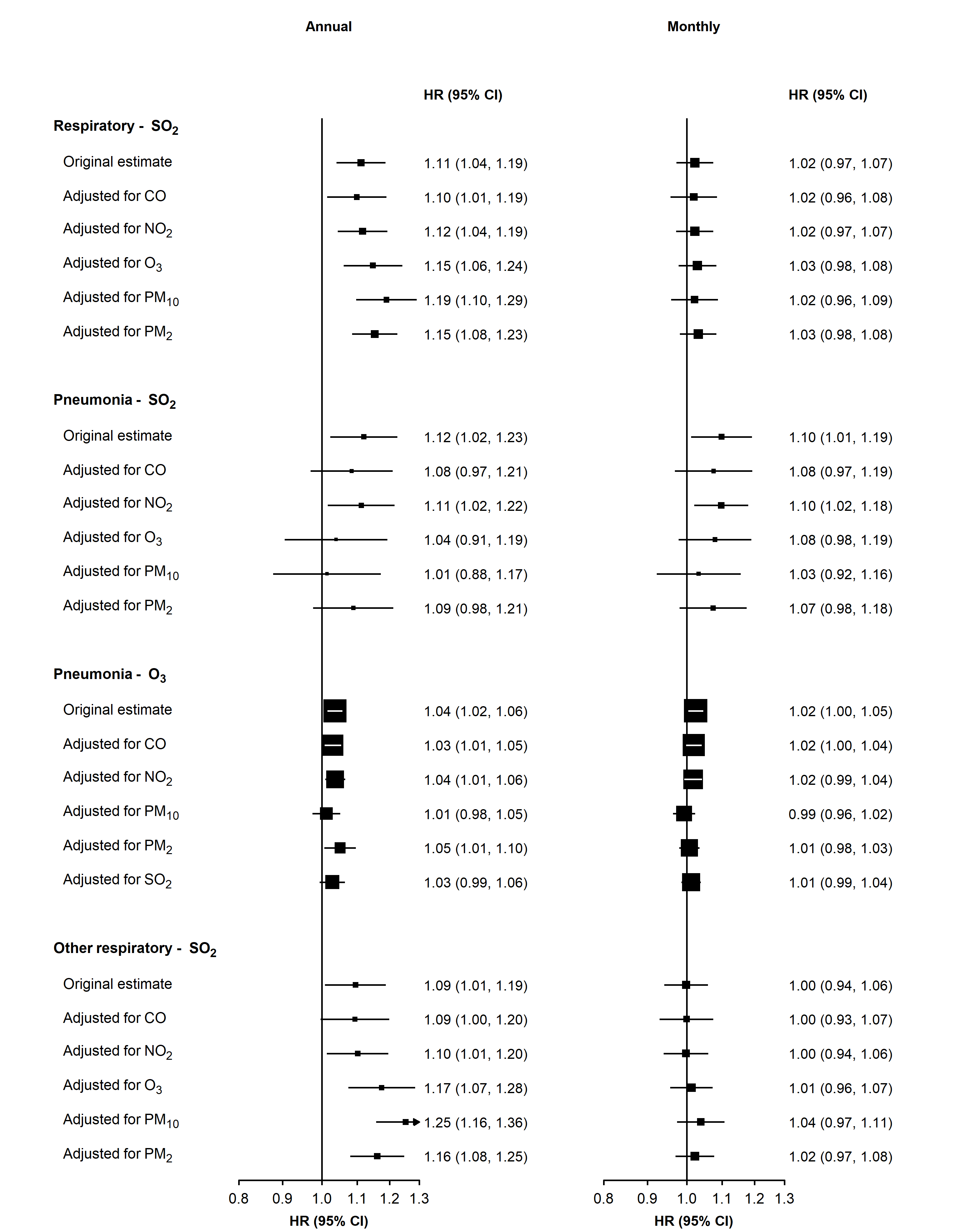


Hazard ratios per 10 µg/m³ increase in pollutant exposure, except CO per 100 µg/m³ increase. Adjusted for age, sex, active smoking status, passive smoke exposure, self-rated health, BMI, total physical activity, alcohol consumption, highest education, solid-fuel use, ambient mean temperature, consumption of fresh fruit and preserved vegetables, diabetes medication, and prior cardiovascular disease.

# eFigure 5: Associations between cardiovascular respiratory diseases and long-term SO2 and O3 exposures using regression splines


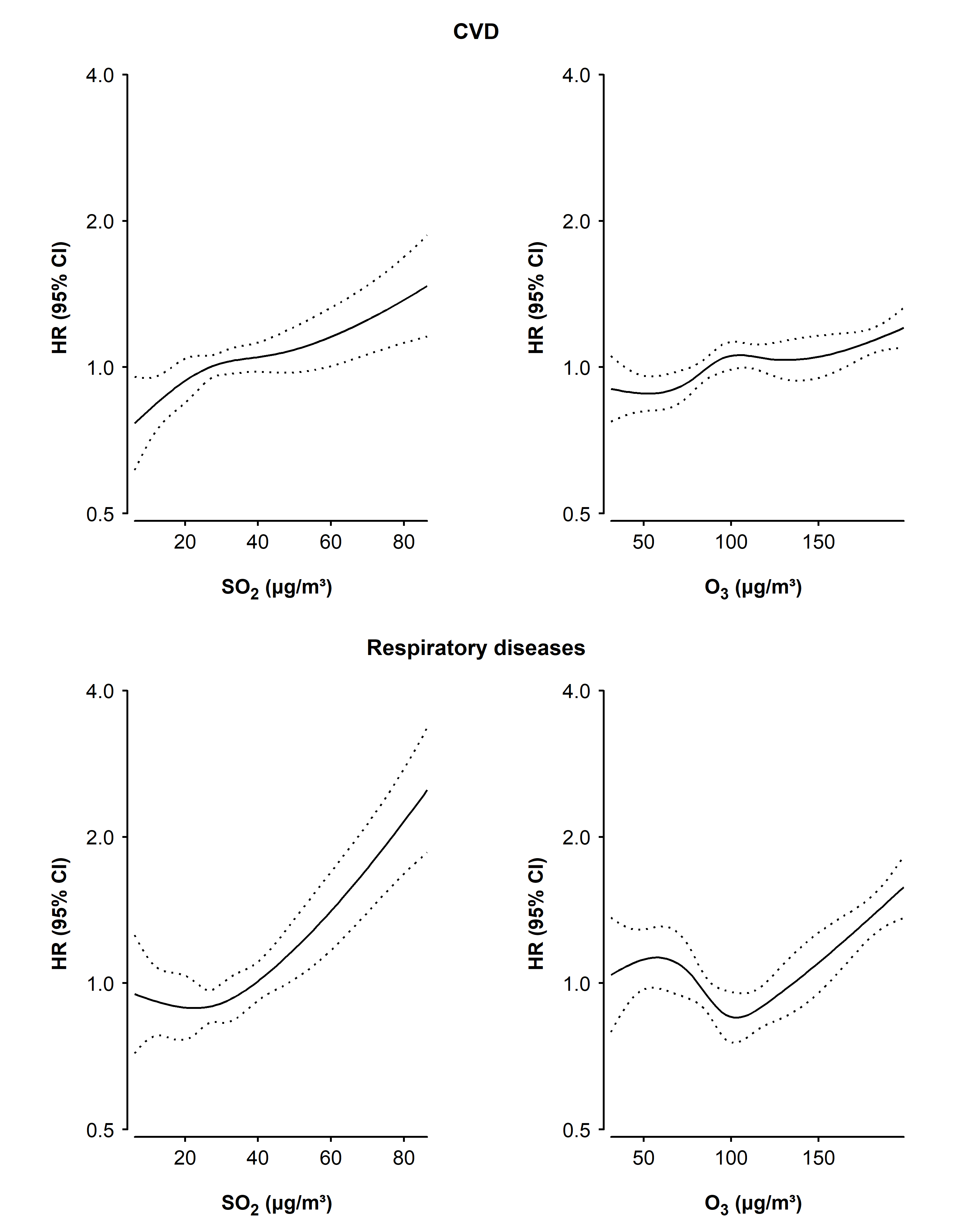


Hazard ratios per 10 µg/m³ increase in pollutant exposure, except CO per 100 µg/m³ increase. All analyses adjusted for age, sex, active smoking status, passive smoke exposure, self-rated health, BMI, total physical activity, alcohol consumption, highest education, solid-fuel use, ambient mean temperature, consumption of fresh fruit and preserved vegetables. Analyses of cardiovascular diseases also adjusted for hypertension, SBP, and prior respiratory disease. Analyses of respiratory diseases also adjusted for diabetes medication and prior cardiovascular disease.

# eFigure 6: Associations between cardiovascular diseases and monthly pollutant exposures


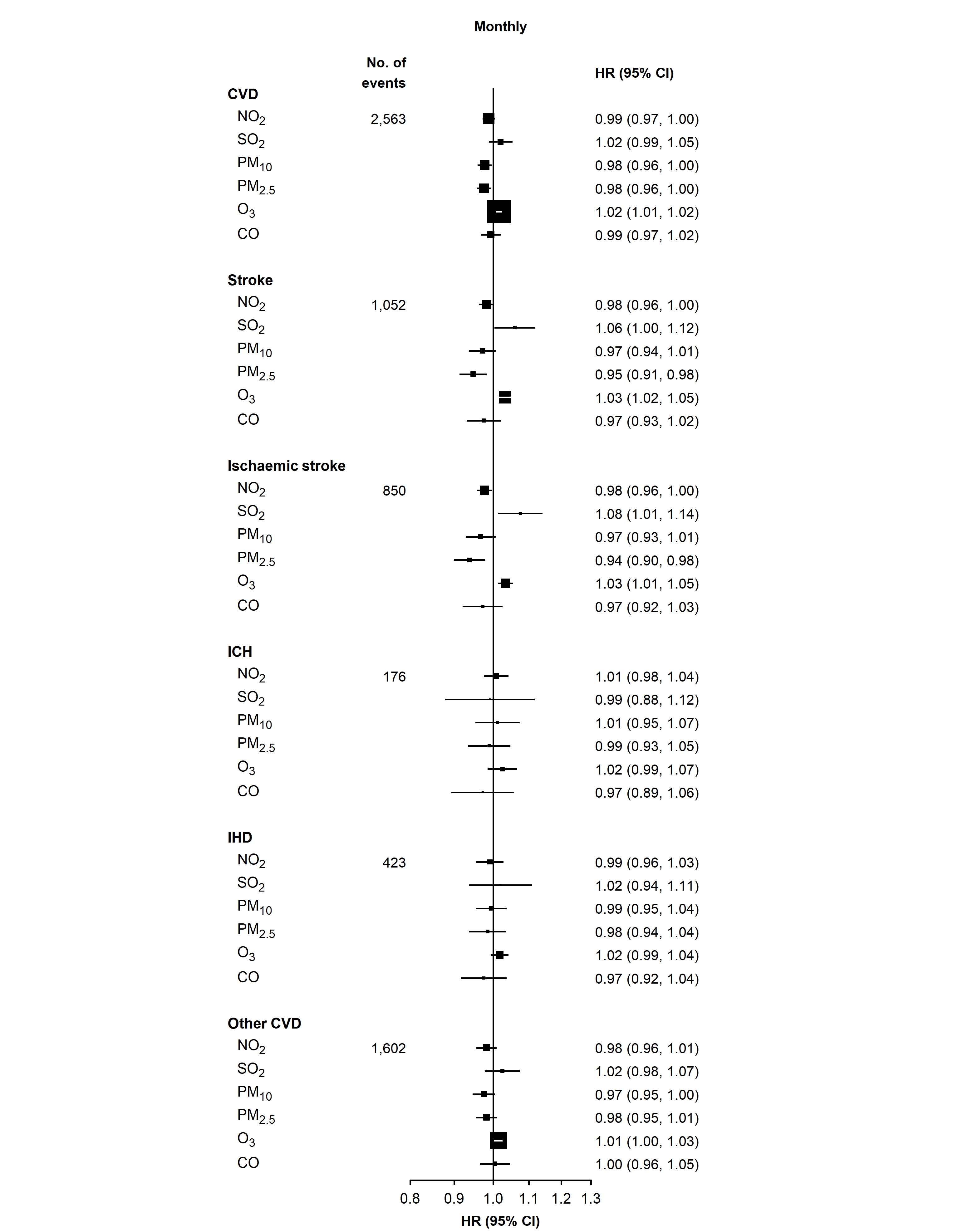


Hazard ratios per 10 µg/m³ increase in pollutant exposure, except CO per 100 µg/m³ increase. Adjusted for age, sex, active smoking status, passive smoke exposure, self-rated health, BMI, total physical activity, alcohol consumption, highest education, solid-fuel use, ambient mean temperature, consumption of fresh fruit and preserved vegetables, hypertension, SBP, and prior respiratory disease.

# eFigure 7: Associations between respiratory diseases and monthly pollutant exposures


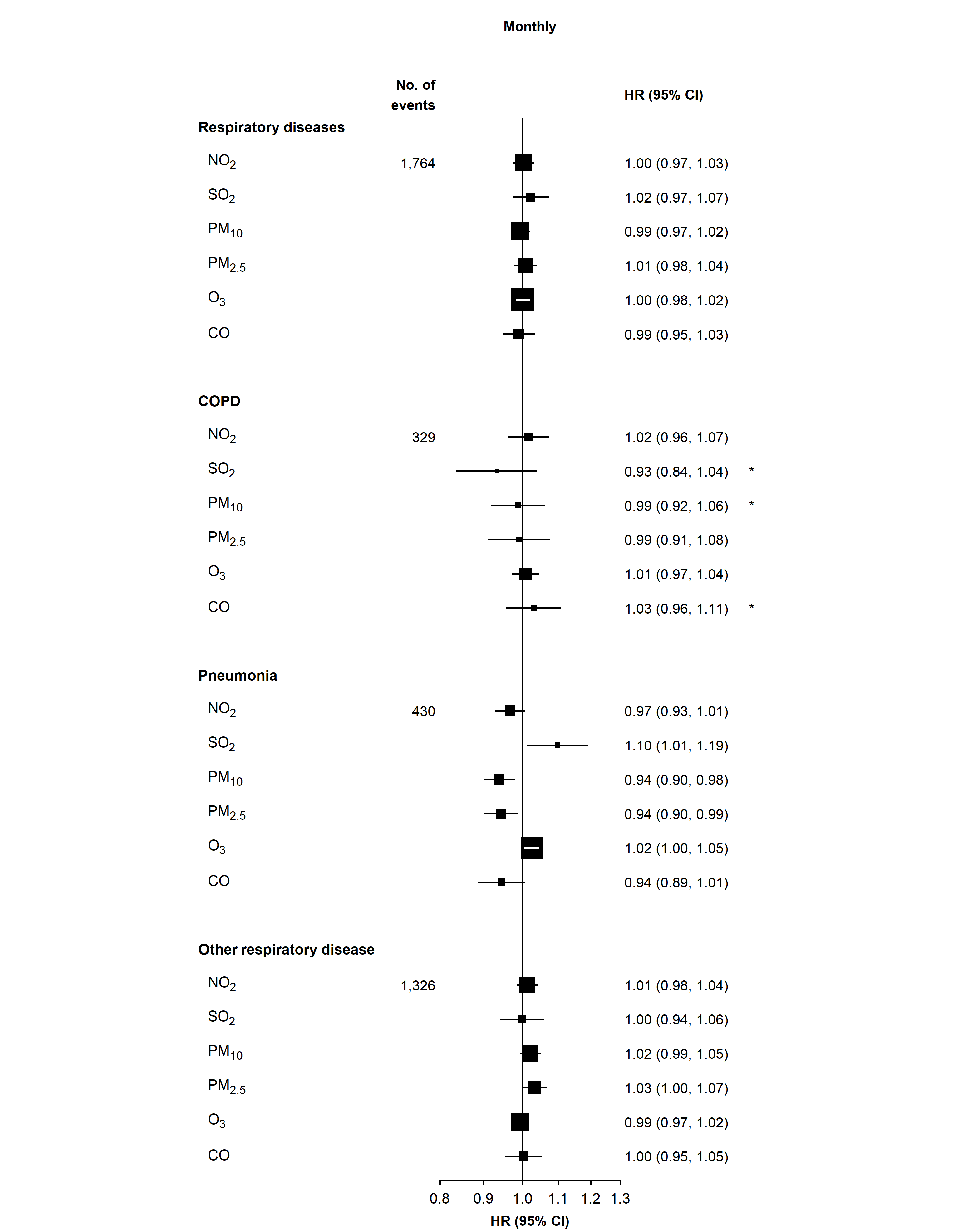


Hazard ratios per 10 µg/m³ increase in pollutant exposure, except CO per 100 µg/m³ increase. Adjusted for age, sex, active smoking status, passive smoke exposure, self-rated health, BMI, total physical activity, alcohol consumption, highest education, solid-fuel use, ambient mean temperature, consumption of fresh fruit and preserved vegetables, diabetes medication, and prior cardiovascular disease.

* HR is not adjusted for temperature, due to problems with model convergence.

# eFigure 8: Associations between cardiovascular disease and long-term pollutant exposures adjusted for different variables


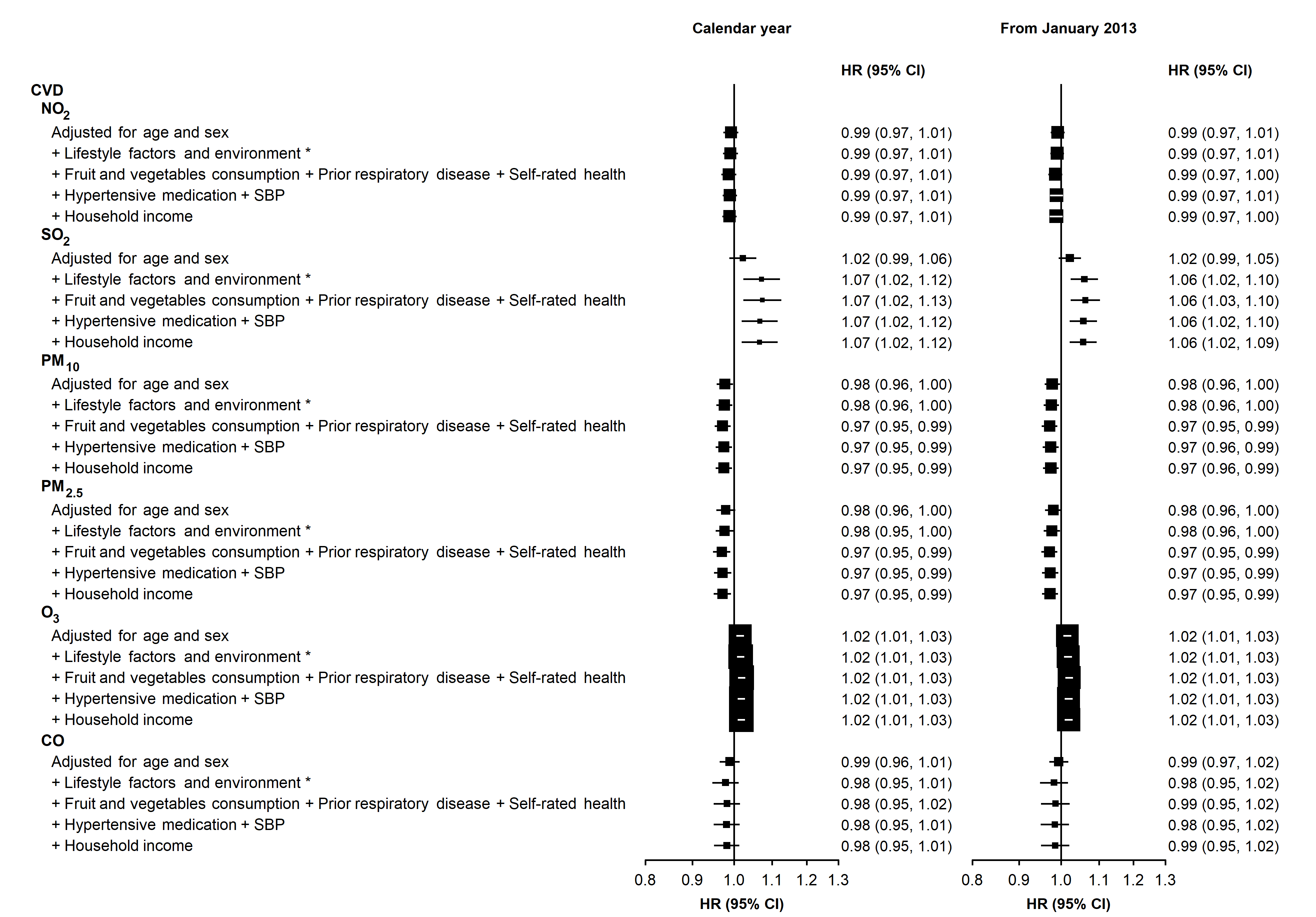


* Active smoking, exposure to passive smoking, body mass index, physical activity level, alcohol consumption, highest education level, solid-fuel use for cooking, and ambient mean temperature.

# eFigure 8: Associations between respiratory disease and long-term pollutant exposures adjusted for different variables


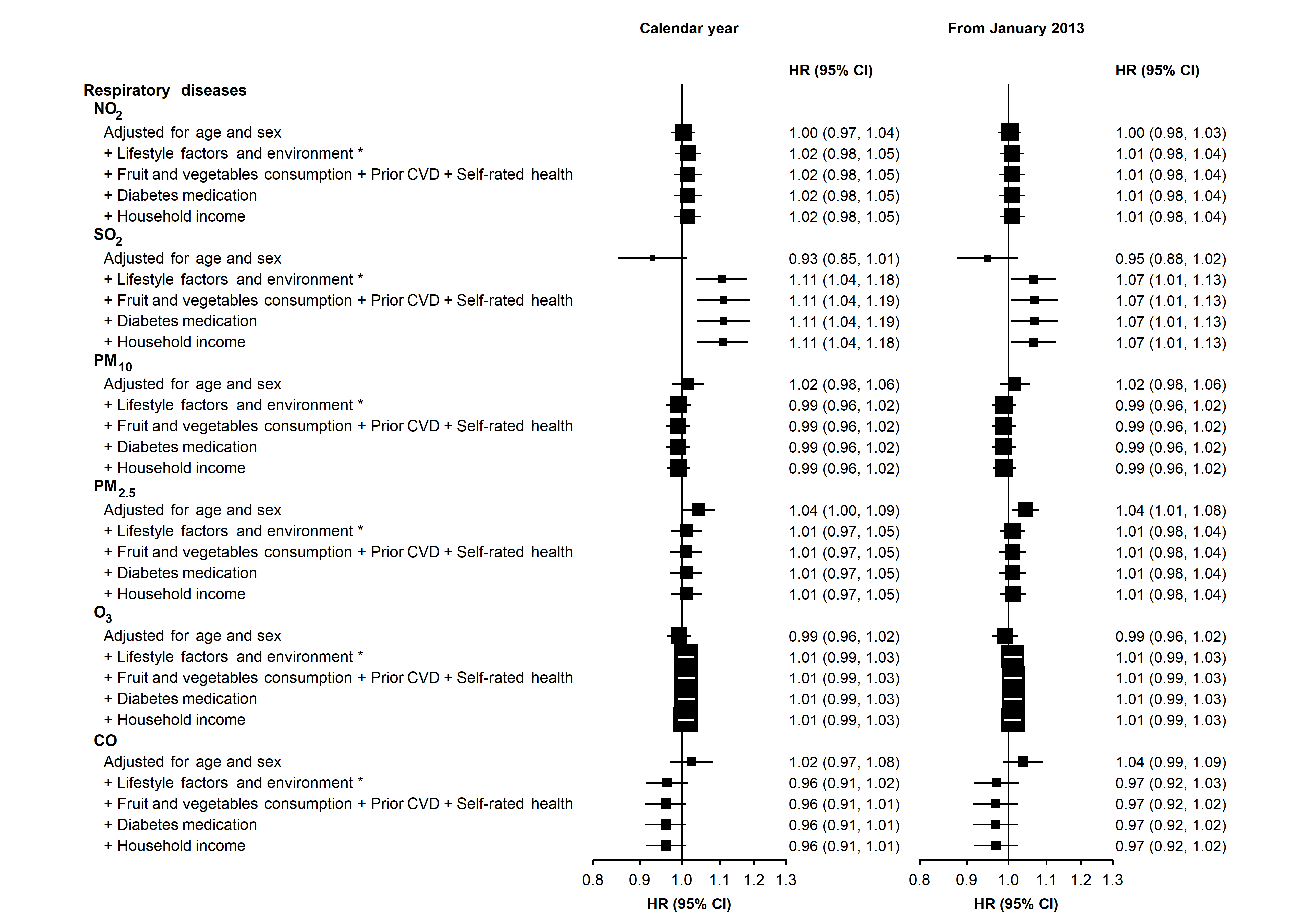


* Active smoking, exposure to passive smoking, body mass index, physical activity level, alcohol consumption, highest education level, solid-fuel use for cooking, and ambient mean temperature.
